# Characterization of Cannabis users and products and the experience of negative mental emotions following Cannabis use

**DOI:** 10.1007/s00406-024-01812-0

**Published:** 2024-06-11

**Authors:** Nir Treves, Noa Yakirevich-Amir, Wiessam Abu Ahmad, Omer Bonne, Elyad Davidson, Keenan Keeling, Branden Hall, Tyler Dautrich, Ilan Matok

**Affiliations:** 1 Division of Clinical Pharmacy, School of Pharmacy, The Hebrew University, Jerusalem, Israel; 2 Department of Psychiatry, Hadassah Hebrew University Medical Center, Jerusalem, Israel; 3 Hebrew University, Hadassah Braun School of Public Health and Community Medicine, Jerusalem, Israel; 4 Department of Anesthesia CCM and Pain Management, Hadassah Hebrew University Medical Center, Jerusalem, Israel; 5 MoreBetter, Ltd. (dba Releaf App), Hyattsville, MD USA

**Keywords:** Cannabis, Medical cannabis, Adverse events, Psychiatric adverse events, Negative mental emotion

## Abstract

There is a potential link between cannabis and mental disorders﻿. Cannabis exposure involves in many cases negative mental emotions, which are unpleasant sensations or thoughts. Whereas mild cases of negative mental emotions inflict patient’s quality of life, more severe cases lead to therapy discontinuations, or even hospitalizations and death. This study characterizes cannabis users who experienced negative mental emotions after cannabis exposure. The Releaf App database was utilized to evaluate the association between personal and cannabis use characteristics on reporting a negative mental emotion during cannabis exposure. This global mobile lets individuals track real-time cannabis experience use with cannabinoid-based products, containing data points such as gender, age, reason*s* for use, product type, cannabis composition, and feelings and emotions experienced after cannabis use. Multivariable logistic regression models were constructed, adjusting for potential confounders such as gender and previous experience with cannabis use. The study population comprised 4,435 users, and 34,279 sessions were collected from various countries, mainly from North America, and included in the primary analysis. Reporting on negative mental emotions was associated with users in the age group of 18–30 years. Using cannabis for a mental purpose was associated with a small increase in reporting on negative mental emotions (OR = 1.10, 95%CI [1.03–1.19]). Oral products were associated with reporting on negative mental emotions. THC-dominant products were associated with reporting negative mental emotions compared to balanced products (OR = 1.21, 95%CI [1.06–1.38]). This study suggests that some characteristics of cannabis use, such as young age and oral consumption are associated with negative mental emotions. Further studies should examine the interface between cannabis consumption, characteristics of consumers, and negative emotional experience or even long-term mental disorders.

**Supplementary Information:**

The online version contains supplementary material available at 10.1007/s00406-024-01812-0.

## Introduction

In the last decades, cannabis for recreational and more so for medical use has become more common than ever. United Nations (UN) reports estimate that over 192 million people globally were exposed to cannabis, reflecting a 4% annual prevalence of the worldwide population [1]. Based on the 2018 Annual Report Questionnaire, the annual prevalence of cannabis use in the United States is estimated as 19.4% in the adult population [2].

The immediate and prolonged effects of cannabis use are varied as it depends on the user’s characteristics such as age, medical history comorbidities, exposure to other drugs, cause for consumption [3–5]. Furthermore, cannabis products are different in tetrahydrocannabinol (THC) and cannabidiol (CBD) composition, other cannabinoid and non-cannabinoid content (such as terpenes), and method of consumption.

Cannabis may have beneficial effects on certain disorders. Many countries formally approved cannabinoids as a treatment for conditions such as epilepsy, nausea and vomiting, and spasticity in multiple sclerosis (Epidiolex, Dronabinol, and Sativex, respectively). Despite limited safety and efficacy data, cannabis is also used for conditions such as post-traumatic stress disorder (PTSD), inflammatory bowel diseases, and pain, designated as "medical cannabis" programs in a growing number of countries [6, 7]

In addition to the attempt to introduce cannabis into the bona fide medical pharmacopeia, cannabis is widely marketed for a wide array of conditions. Thus, online cannabidiol retailers in Canada claimed efficacy and effectiveness for cannabis in 171 conditions. Some of these are for medical conditions such as multiple sclerosis, anxiety, epilepsy, and cancer, while others are less for medical entities and primarily serve to relieve mood, fatigue, discomfort, and mental unclarity [8].

Although numerous cannabis products are marketed for emotional, mental, or mental-related conditions, one of the main concerns of cannabis use is its potential to cause or exacerbate mental and psychiatric disorders. Various reports and studies found an association between the use of cannabis and the new onset or exacerbation of mental conditions, specifically psychotic disorders [9–11]. Di-Forti et al. reported that daily exposure to cannabis was associated with higher rates of psychotic disorders compared with those who reportedly never used cannabis (OR = 3.2, 95%CI 2.2–4.1) [12]. In addition, cannabis was also reportedly associated with events of anxiety, dissociation, and mood changes [13]. However, it is still unclear whether one-time cannabis or chronic cannabis use may trigger substantial long-term mental deterioration rather than temporary toxification [12, 14–17]. Contrarily, others argue that cannabis may alleviate mental distress and even psychotic symptoms [18], including improvement of psychotic symptoms in schizophrenia [19].

Milder cases of adverse reactions to cannabis exposure are characterized by negative mental emotions, which are unpleasant, and often transient, sensations or thoughts, such as a state of confusion, disorganized thoughts, anxiety, slow responsiveness, and even paranoia [20]. Negative mental emotions are associated with commonly used medications as well such as antiepileptics [21], antiviral agents [22], and even antidepressants [23]. In the settings of these triggering treatments, while mild cases of negative mental emotion inflict the patient’s quality of life, more severe cases lead to therapy discontinuations [24], or even cause hospitalizations and death [25].

Given the paucity of data, we wondered whether the outcome of cannabis usage may be, at least in part, dependent upon the characteristics of users and the reason for its consumption. We believe an association between personal and sociodemographic characteristics and the effects of cannabis on emotional and mental well-being should be explored. This study characterizes consumers who experienced negative mental emotions a short time after cannabis exposure, utilizing data from a self-reported real-time database.

## Methods

In this naturalistic observational study, we used self-reported experiences of cannabis users from the database of the Releaf App™ educational mobile software. This global mobile and tablet app lets users track sessions and real-time cannabis user experience and collects retrospective real-world data on an individual’s use and experience with specific cannabinoid-based products. The freely available in English for both Android and iOS devices. Given the relatively scant scrutiny and regulatory oversight applied to cannabis products, the app serves a pivotal role in bridging this gap, as it facilitates the users to document their cannabis use aiming to improve their use of cannabinoid based products. It has been used by state government agencies to inform policy decisions. It has been used by federal government agencies to access Real World Data on the use of cannabinoid products in a wide population of patients and consumers, and was previously utilized as a database in more than a dozen published studies.

Registration to the app is free and subject to the user’s consent to use the app registry as a data source for research purposes after anonymization to maintain users’ privacy. Since user consent was acquired during app registration and due to the anonymized nature of the study, no further ethics approval was required for execution of this study. The de-identified, app-user-level data were provided to the research team by the owner of the Releaf App, MoreBetter, Ltd. under a data confidentiality agreement. The data collected in the Releaf App is MoreBetter’s property. Therefore, access to the data availability and the coding for processing is limited and requires contacting and the consent of MoreBetter. The coding used for the statistical analysis of this study is published in Appendix 1.

### Study population

The app users are older than 18 years of age, based on self-report filled from February 2016 until June 2021. The app user may report every session of cannabis use and can contribute more than one session to the analysis, and therefore, a mixed effects model was utilized, as described in the section below. The user may also report his experience for each session, mainly done a few hours after the session started. The report includes data about demographic features such as age, gender, and country of living; cannabis use characteristics, including cannabis use experience, the reason provided for cannabis use, product type; and product content based on the ratio of THC:CBD. Most of the cannabis use characteristics may change in different sessions within the same user. In most sessions the user also reported the ranking symptom severity of the reason provided for cannabis, and the putative change in severity after cannabis consumption.

### Case definition in the primary analysis

The Releaf App database comprises items from a predefined list of 47 cannabis-related events, including possible positive, negative, and context-specific cannabis-use-related experiences (See Appendix 2). App users document their experiences using these items. Sessions were grouped into cases and controls. Out of the 47 possible experiences listed, we chose those that indicate a disturbance in emotional or mental status. These include distraction, paranoia, anxiety, scatteredness, restlessness, irritability, or confusion. A session in which at least one or more of these experiences were reported was considered a case session. A session with none of the selected case items was considered a control session. The reporting of a negative mental emotion in a session was defined as the primary outcome. In contrast, the secondary analysis compared the change in severity of pain and mental symptoms if negative mental emotion was reported during the session.

### Exposure groups in the primary analysis

We analyzed the association between age groups, product type, the dominance of the product, and the reason provided for cannabis use and reporting negative mental emotion in a separate multivariate model. Each analysis was built based on the relevant variables, assigned as confounders based on a directed acyclic graph (DAG) (See Appendix 3). In the analysis of the reasons provided for cannabis use, we classified sessions into three groups: pain, mental purpose, and other reasons for use.

### Included covariables

The analysis based on the reported data included *all* the following covariables: age group (divided into groups of ten years), gender (female, male, or other), continent, degree of experience with cannabis use estimated by the user (beginner, user with a little experience, user with a lot of experience, and experts), the reason provided for cannabis use (pain, mental or other purposes, as sorted by experienced psychiatrists and detailed in Appendix 4), and the product type (flowers, vaping concentrates, tinctures, edibles, and pills). The composition of the cannabis product was estimated by the ratio of THC:CBD, as THC-dominant (ratio of at least 1.2:1 in THC:CBD concentration), CBD-dominant (ratio of at least 1.2:1 in CBD:THC concentration) or balanced (ratio of less than 1.2:1 between the two substances).

### Secondary analysis outcome

For most participants, the available data included a ranking of the symptom severity of the reason provided for cannabis use before and after cannabis exposure. Based on this data, we evaluated whether reporting negative mental emotion affects the change in severity of symptoms. The analysis was conducted in the whole population and in the two subgroups of users who utilized cannabis for pain or mental purposes.

### Statistical analysis

Descriptive statistics were utilized for presenting the Sociodemographic characteristic traits of cannabis users and products included in the study: mean and standard deviation were calculated for continuous variables, and distributions were created for categorical variables. We used the “pandas” package in Python via the platform Jupyter Notebook [26, 27].

In the primary analysis, multivariable logistic regression models with mixed effects were constructed to study the associations between the users’ attributes (age and the reason provided for cannabis use), and cannabis product attributes (product type and product composition), and reported negative mental emotions using the “glmmTMB” package in R programming language [28]. Each model corresponded with its relevant confounders based on DAG to avoid the “Table 2 fallacy”. The “Table 2 fallacy” occurs when multiple effects are estimated and interpreted by a single multivariable analysis, equating the primary and secondary effects in the analysis and giving the reader the misimpression of parity across the effect estimates [29, 30]. As offered by Westreich et al. DAGs were used to draw plausible causal association in the model variables while focusing on the analyzed primary effects [30]. The user was defined as a random effect, and other covariables were defined as fixed effects. Changes in the reason provided for cannabis use, the product type, and composition of products are plausible in different sessions retrieved from the same patient. All these variables were included as fixed effects in the mixed effects model, as they can explain changes in the response variable in the same user.

In the secondary analysis, a linear regression model with mixed effects was constructed to assess the change in the severity of mental and pain symptoms, which were the reason for cannabis use, using the “lme4” package in R programming language [31], between those who reported on a negative mental emotion and those who did not report on such emotion. Outputs are presented as adjusted Odds Ratios (OR) for the primary analysis, and adjusted change in the rank of severity symptom and their correspondent 95% confidence interval (CI) [32, 33].

## Results

**Fig. 1:**
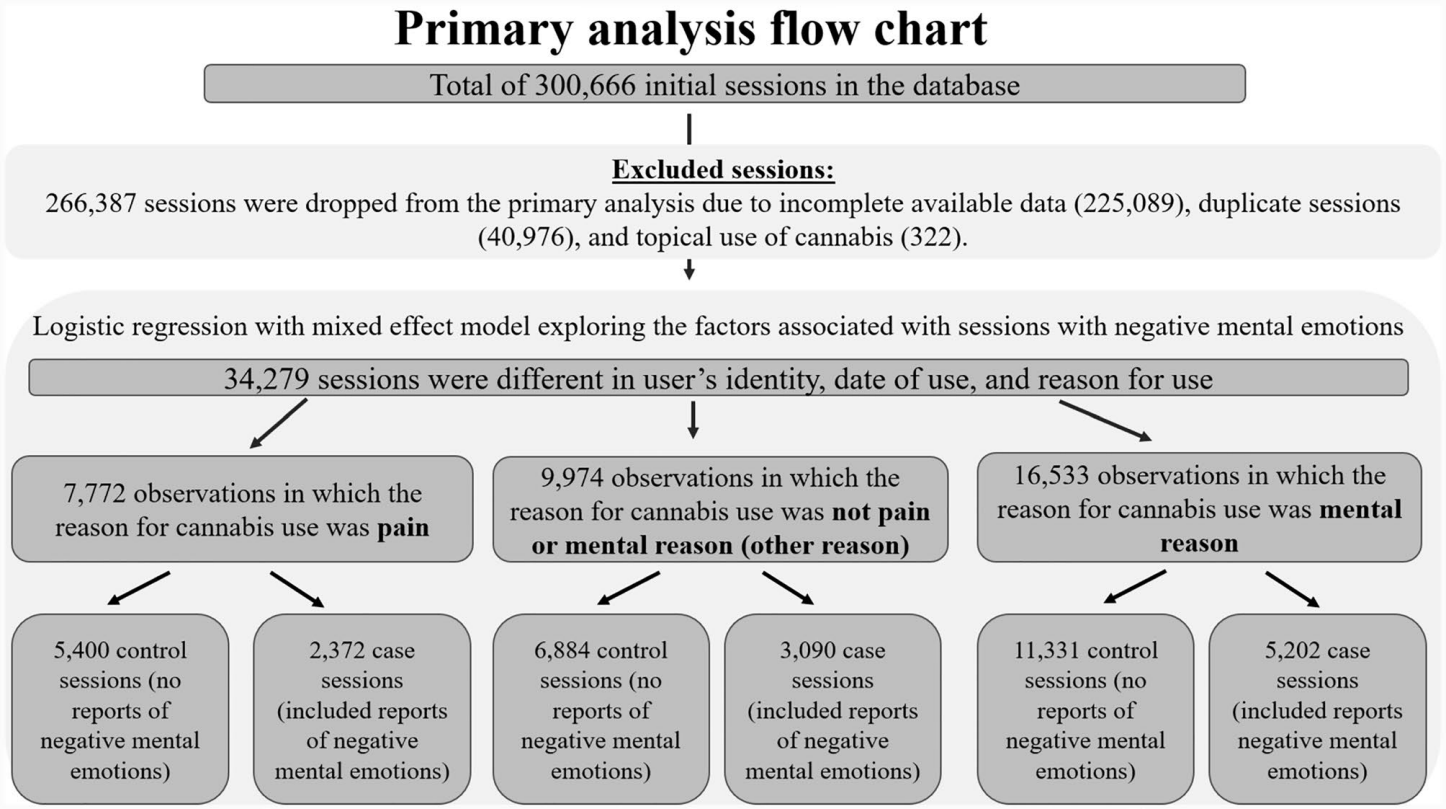
Data extraction of Releaf database – Flow chart of the primary analysis.*Duplicated sessions were omitted since they were identical sessions in terms of user’s identity, date of use, reason for use, and could not contribute more than one time to the analysis

**Fig. 2:**
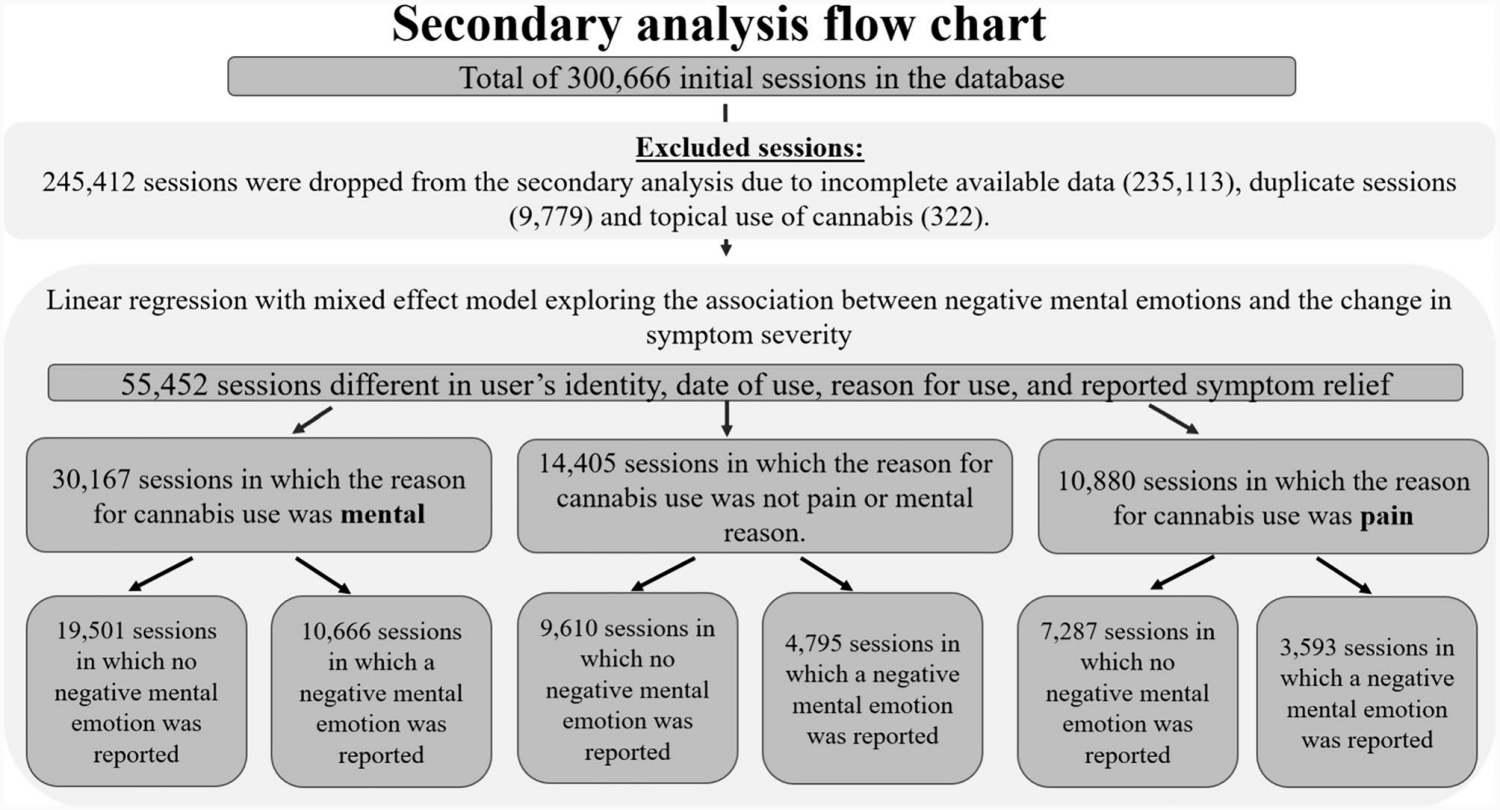
Data extraction of Releaf database – flow chart of secondary analysis. *Duplicated sessions were omitted since they were identical sessions in terms of user’s identity, date of use, reason for use and ranking symptom relief and could not contribute more than one time to the analysis (for more explanation about the differences between the primary and secondary flow charts see Appendix 5)

In the primary analysis, the study population comprised 4,435 users, who reported 34,279 sessions different in date of use and containing complete data regarding their cannabis experience (Fig. 1). 55,452 sessions were included in the analysis for the secondary outcome assessing symptom relief in case of reporting negative mental emotions (Fig. 2).

Table 1 presents the characterization of the sessions in the primary analysis. Users aged 18–30 reported less than 25% of the sessions, those aged 30–40 reported about a third, the age group of 40–50 years reported about 25% of the sessions, and older users reported about 20%. Almost 60% of the sessions were reported by those who defined themselves as beginners or with little experience with cannabis while the remaining roughly 40% of the sessions were reported by those who defined themselves as experts or have a lot of experience. Approximately 84% reported that they used a product administrated via the lungs (smoking or vaping with concentrates and flowers), whereas the remaining 16% used an oral product (edibles, pills and tinctures). In 77.1% of the sessions, the users consumed THC-dominant products, while the rest were split roughly equally between balanced and CBD-dominant products. Similar observations are found in user distribution (Table S1). Of 34,279 sessions, 10,664 negative mental emotions were reported (31.1%). About 27% of the negative mental emotions were reported by users under 30 years of age, representing a slightly higher portion than its share in the total number of sessions in the study population (Table S2).

**Table 1:** Characterization of included sessions in the study

Session characterization	N	% out of total
Age group (Years)		
18–30	8,318	24.3
30–40	10,699	31.2
40–50	8,446	24.6
50–60	4,498	13.1
60–70	2,038	6.0
70–90	280	0.8
Continent		
North America (United states and Canada)	33,313	97.2
Europe	554	1.6
Asia	7	0.02
America American countries not including United Stated and Canada)	223	0.7
Africa	65	0.2
Australia	117	0.3
Gender		
Female	19,897	58.0
Male	13,132	38.3
Other	1,250	3.7
Experience		
Beginner	5,150	15.0
Little experience	14,651	42.8
A lot of experience	10,344	30.2
Expert	4,134	12.0
Product Type		
Concentrate	12,085	35.3
Edible	2,247	6.6
Flowers	16,644	48.6
Pill	629	1.8
Tincture	2,674	7.8
Dominance		
THC	26,435	77.1
Balanced	3,292	11.2
CBD	4,552	12.6
Reason provided		
Pain	7,772	23.4
Mental reason	16,533	47.6
Other reason	9,974	29.0
Cases		
Negative mental emotions were reported during the session	10,664	31.1

**Fig. 3:**
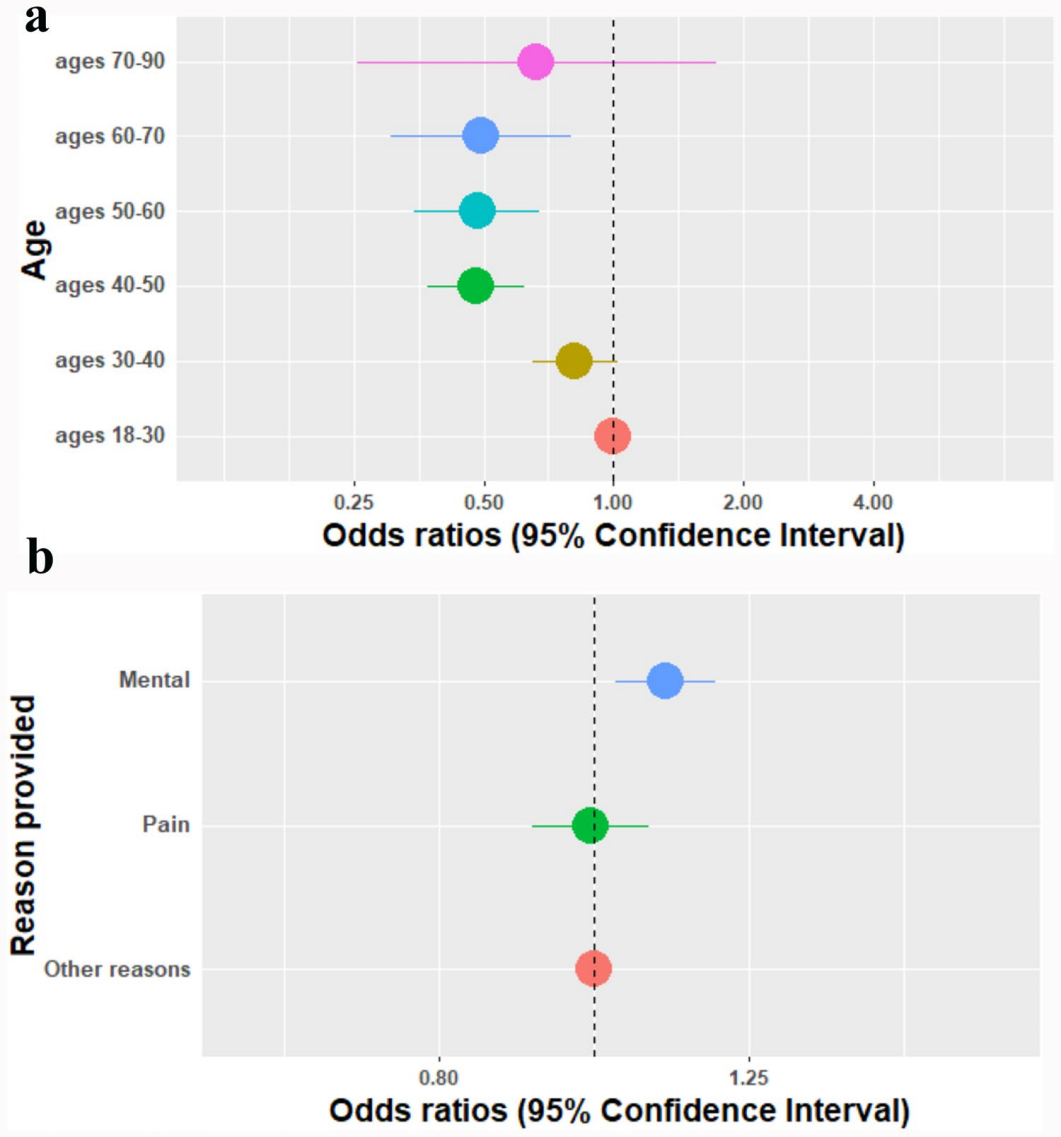
**a** The association between age and reports of negative mental emotions. *Adjusted for gender, experience, and the reason provided. **b** The association between the reason provided for cannabis use and reports of negative mental emotions. *Adjusted age, gender, experience, dominance, product type

*The association between users’ attributes age and reason for use and negative mental emotions*: Reporting on negative mental emotions was associated with younger ages, as the odd ratios of 18–30 years were higher than most other age groups (OR = 0.82, 95%CI [0.65–1.02], OR = 0.48, 95%CI [0.37–0.62], OR = 0.48, 95%CI [0.35–0.67], OR = 0.49, 95%CI [0.30–0.80], OR = 0.66, 95%CI [0.25–1.73],in the age groups 30–40 years, 40–50 years, 50–60 years, and 70–90 years compared with 18–30 years, respectively) (Fig. 3a). No interactions were found between age and gender for reporting negative mental emotions (Appendix 6).

Using cannabis for a mental purpose such as depression or anxiety was associated with a small increase in reporting on negative mental emotions, whereas using cannabis for pain was not associated with reporting negative mental emotions (OR = 1.10, 95%CI [1.03–1.19], OR = 0.99, 95%CI [0.91–1.08], compared to other symptoms) (Fig. 3b).

**Fig. 4:**
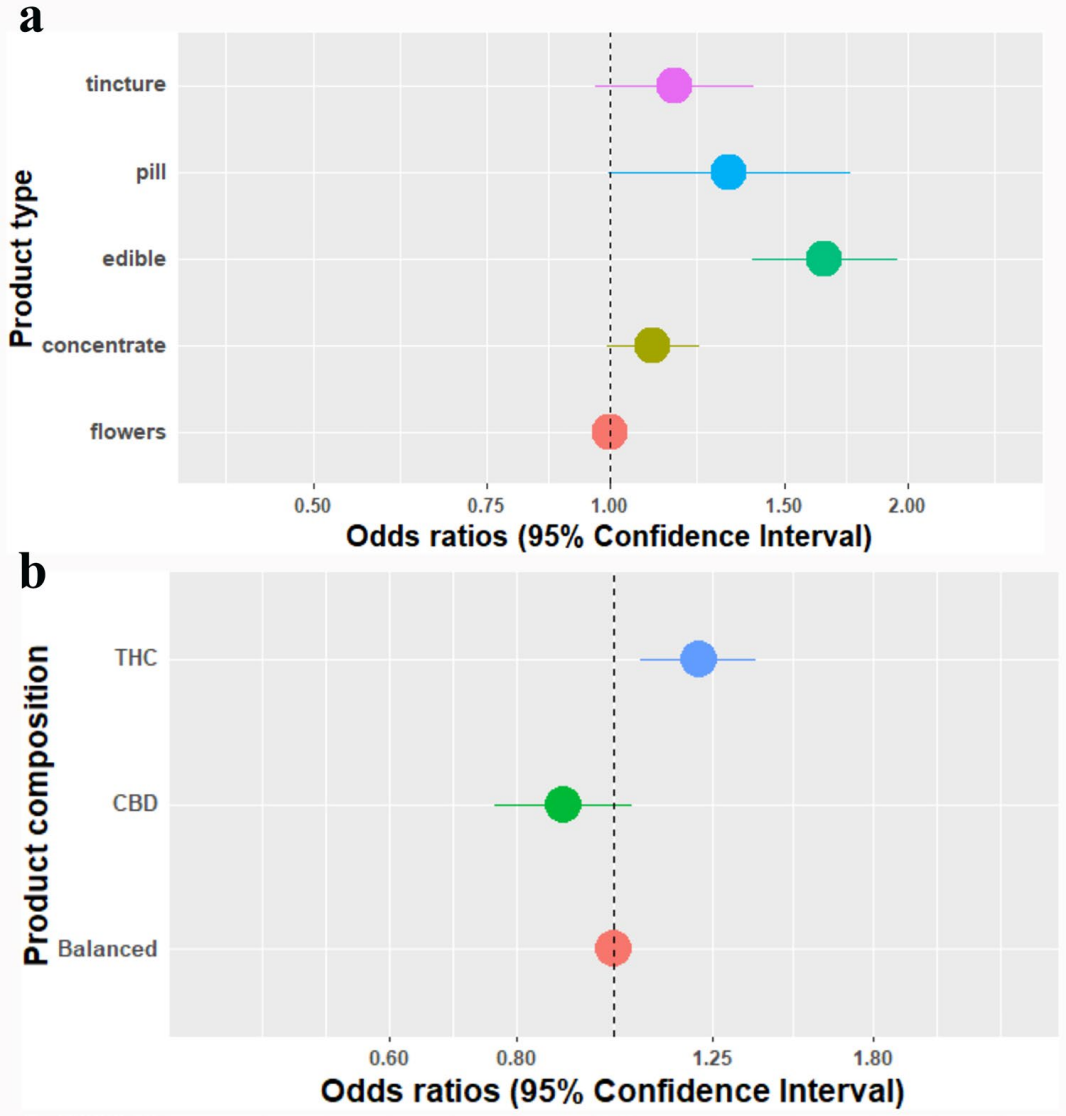
**a** Product types in users who experience negative mental emotions. Adjusted to reason provided for use, product composition, and experience. **b** Cannabis composition in users who experience negative mental emotions: *Adjusted to reason provided for use, product type, and experience

*Odds ratios for negative mental emotions – the association with cannabis product attributes:* Product types were associated with negative mental emotions, including concentrates and oral products (OR = 1.10, 95%CI [0.99–1.23], OR = 1.16, 95%CI [0.97–1.40], OR = 1.65, 95%CI [1.39–1.95], OR = 1.32, 95%CI [1.00–1.75] in concentrates, edible products, pills, and tinctures respectively compared to flowers) (Fig. 4a). THC-dominant products were associated with reporting more negative mental emotions compared to balanced products (OR = 1.21, 95%CI [1.06–1.38]) (Fig. 4b).

**Fig. 5:**
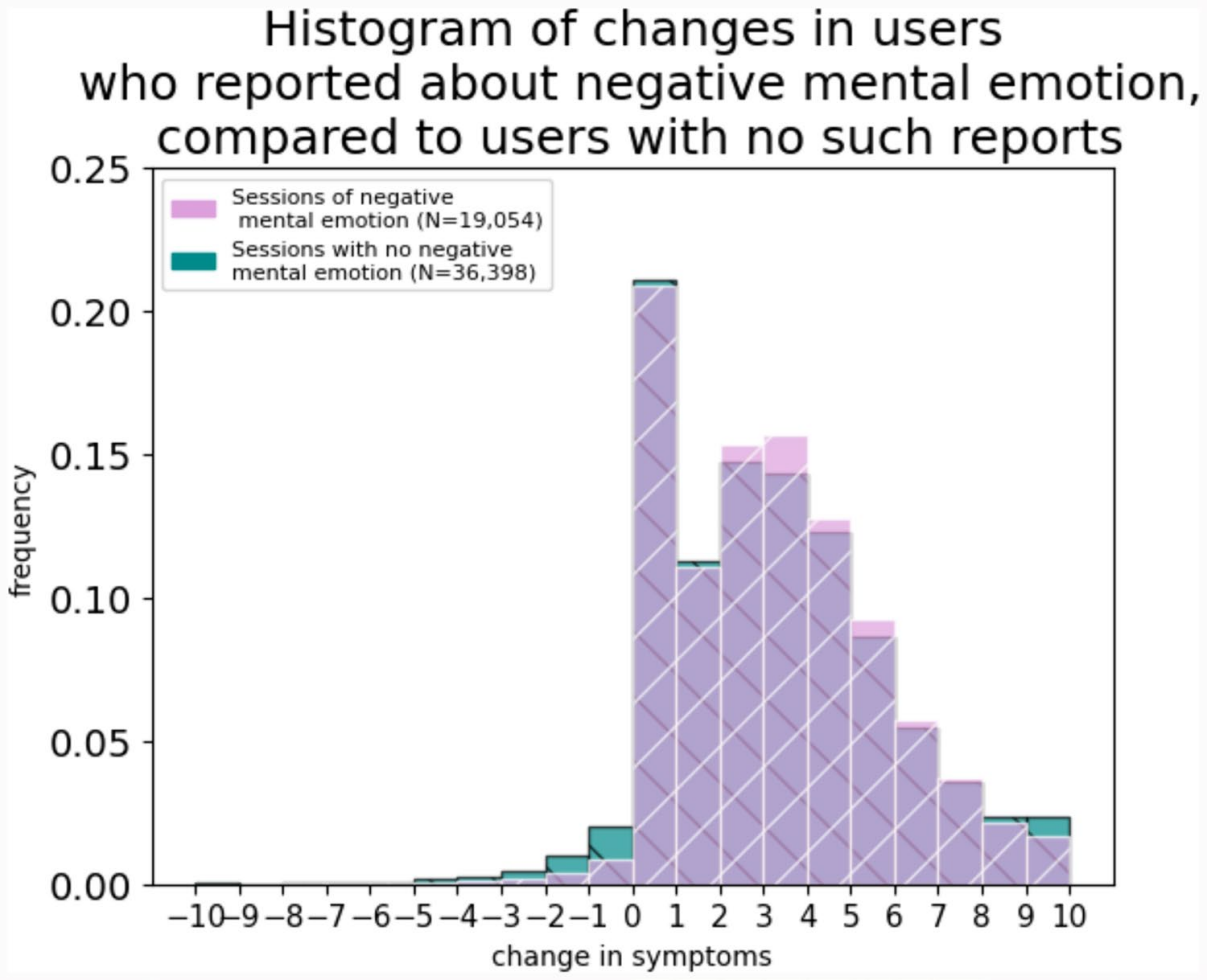
Changes in symptoms in users who reported negative mental emotions compared to users with no such reports

*Changes in symptoms in sessions with negative mental emotion:* We also evaluated the change in symptoms for which cannabis was used in those who reported a negative mental emotion while adjusting to other covariables with linear regression. Based on the analysis, after adjusting to covariables in a mixed effect model, reporting on negative mental emotion was not associated with worsening or improvement in the symptom average (Fig. 5). In the subgroup analysis in pain or mental users, no such effect was observed as well (0.02, 95%CI [ – 0.02 to 0.07]; 0.02, 95%CI [ – 0.05 to 0.09], 0.02, 95%CI [ – 0.03 to 0.08], respectively).

## Discussion

To our knowledge, it is the first observational study on large scales assessing the characteristics of cannabis users who experience a negative mental or emotional effect, based on real-time reporting data. In the last decades more countries allow cannabis use for various medical indications and legalize cannabis recreational use, and thus the putative risk of cannabis use must be determined. If cannabis use is indeed found hazardous, it is imperative to learn whether certain characteristics predispose individuals to the negative attributes of this substance [12, 34].

Although most of the data was collected from North America, it represents general trends of cannabis utility in Western societies, including Europe, and focused on cannabis utility attributes rather than the consumption of specific products marketed only in North America. Many markets around the world look at the developments in the United States to better inform how to approach medical cannabis legalization and access. Given this focus, the findings have merit. They can help inform patients, healthcare providers, regulators, and researchers at a global level, specifically in Europe, where approximately 8% of European adults (22.6 million aged 15 to 64) have used cannabis in the last year. These results are heterogeneous at the national level, probably due to different approaches for the medical and legalized cannabis use [3, 35, 36].

In this study, most of the participants were females. This distribution was seen in previous studies based on this database [37], but conflicts with known data suggesting that cannabis consumption among men is more frequent [38]. Nevertheless, cannabis use among women is increasing, and recent studies based on validated registries demonstrated a majority of females for cannabis use [39, 40]. Nevertheless, the high prevalence of women in our database most likely reflects differential reporting habits between genders.

This study suggested that specific characteristics of cannabis users are associated with more reports of negative mental emotions, such as young age (< 30 years). Regarding the cannabis product, the oral route of consumption was associated with more reports of negative mental emotions than the inhaled route of consumption, as well as mental reasons for cannabis use and THC-dominant products. This work did not include the negative mental emotions in children, although previous work showed that cannabis treatment was associated with negative cognitive and mental emotions in this population [41].

Most mental disorders appear before age 35 and reckless cannabis consumption also reduces with age [42, 43]. The notion that older individuals are less mentally triggered by cannabis, can be linked to the mixed evidence of the changes in the endocannabinoid system during aging. Some *in-vivo* studies suggest a reduction in the endocannabinoid tone in aging, expressed mainly by the decline of CB1 receptors in several regions in the brain, namely the cortex, the cerebellum, the hippocampus, and the limbic and hypothalamic structures [44]. On the other hand, other works did not find a low density of CB1 in the older murine CNS but indeed reported diminished coupling between CB1 receptors and Gi proteins, leading to diminished cannabinoid signal transduction [45]

Most oral cannabis products were associated with a substantially increased risk for negative mental emotions compared to smoking flowers. THC oral ingestion leads to extensive metabolism in the liver of the prominent psychoactive metabolite of THC, 11-OH-THC, which may be responsible for some of the cannabis effects, and one of them might be the induction of negative mental emotions [46]. Oral administration of THC was also reported to induce anxiety specifically in naïve subjects rather than experienced users who are more familiar with cannabis [46]. On the other hand, the study cannot exclude a reverse causal where users with a predisposition to negative mental emotions after cannabis exposure tend to consume oral cannabis.

THC-dominant products were reported to be associated with the risk for negative mental emotions, compared to balanced and CBD-dominant products. These results corroborate the results of previous studies that reported an elevated risk of psychosis after THC exposure, which is known for its varied psychoactive effects [12]. Nevertheless, it is noteworthy that the obtained data only contained information regarding the THC and CBD levels in the product, while the dose, the total THC concentration and the contents minor cannabinoids were not introduced to the analysis due to lack of data.

The study has several limitations. First, it is an observational study, and it is hard to determine whether the app users represent the population who consume cannabis. The outcomes are based on self-reports and are not considered medically validated tests for psychiatric assessment. Nevertheless, the negative mental emotions reported by users indicate a specific state of mind, which may be unpleasant in itself. In addition to reflecting the momentary experience of the cannabis user, whether this is an indicator or a risk factor for the evolution of a more severe and enduring mental condition remains open. While cannabis is gradually more accepted as a medical therapy, its association with various emotional and psychiatric effects should be considered, since it may affect the adherence and safety profile of the treatment, like in other psycho-affective therapies [21, 22, 47].

Another area for improvement relates to the knowledge and skills of users. Although the use of the app is intuitive, it requires the following essential skills and knowledge, which may introduce a bias: minimal familiarity with cannabis products, to identify the active ingredients THC and CBD, basic technology orientation to operate the app, and basic knowledge of English, since it is only available in English.

However, these requirements are trivial for most users. Medicinal and legalized cannabis programs require full label details on the product at licensed dispensaries as required by law. Therefore, as long as cannabis use is within the law, this info is always available to the users who can fill this data manually into the Releaf App. In some cases, the product label detail is pre-populated. In addition, the study utilized only basic data about the THC:CBD ratio, and therefore, it is highly conceivable that the Releaf app users are familiar with this consumption level. In addition, the app is currently only available in English, so there might be a selection bias.

In addition, several personal and use characteristics were unavailable, such as whether the cannabis use is medical or recreational, the exact dose taken by the user, comedications, and comorbidities. In addition, the mental state of the users at baseline is unknown. Although some users state that the reason they consume cannabis is self-medication, their actual mental condition is not known. It is essential to investigate further whether those in compromised mental states are at more risk for negative mental emotions after cannabis consumption.

## Conclusions

Albeit its limitations, this is the first study based on a large-scale, real-time database acquired during cannabis consumption examining an association between cannabis use and negative emotional symptoms. It suggests that some characteristics of cannabis use, such as oral consumption and young age, are associated with negative emotion symptoms that can inflict cannabis users and cannabis patients. Cannabis use for mental reasons was associated with more negative emotional experiences than use for pain user. Further studies should examine the potential interface between cannabis consumption, characteristics of consumers, and negative emotional experience or perhaps even long-term mental disorders.

## Electronic supplementary material

Below is the link to the electronic supplementary material.

Supplementary file1 (DOCX 19 KB)

## Data Availability

The data used in this study cannot be shared publicly as it belongs to MoreBetter Ltd. However, the code used for analysis and implementation is available in the supplementary materials accompanying this paper. Researchers interested in accessing the raw data may contact MoreBetter directly for permissions and access.

## Appendices

See below Appendices 1, 2. 3, 4, 5 and 6.

## Appendix 1

R script for statistical analyses

#DAG

Library(dagitty).

#data for creating the DAG plot.

g1 <—dagitty( "dag {

age—> negative_mental_feeling.

product_type <—dominance—> negative_mental_feeling.

reason_provided—> product_type—> negative_mental_feeling.

age <—gender—> negative_mental_feeling.

reason_provided—> negative_mental_feeling.

reason_provided <—age.

reason_provided <—gender.

reason_provided—> dominance.

experience—> negative_mental_feeling.

reason_provided <—> experience.

experience—> product_type.

experience—> dominance.

}")

#creating the plot.

plot(graphLayout(g1)).

#Sets for adjustment based on the DAG.

print( adjustmentSets( g1, "age", "negative_mental_feeling",effect = "direct")).

#{ experience, gender, reason_provided}.

print( adjustmentSets( g1, "reason_provided", "negative_mental_feeling",effect = "direct")).

#{ age, dominance, experience, gender, product_type}.

print( adjustmentSets( g1, "product_type", "negative_mental_feeling",effect = "direct")).

#{ dominance, experience, reason_provided}.

print( adjustmentSets( g1, "dominance", "negative_mental_feeling",effect = "direct")).

#{ experience, product_type, reason_provided}.

#Primary analysis, random effects logistic regression.

Primary_analysis29Nov22_test2$age_cat = as.factor(Primary_analysis29Nov22_test2$age_cat).

Primary_analysis29Nov22_test2$dominance = as.factor(Primary_analysis29Nov22_test2$dominance).

Primary_analysis29Nov22_test2$gender_simple = as.factor(Primary_analysis29Nov22_test2$gender_simple).

Primary_analysis29Nov22_test2$experience = as.factor(Primary_analysis29Nov22_test2$experience).

Primary_analysis29Nov22_test2$product_type = as.factor(Primary_analysis29Nov22_test2$product_type).

Primary_analysis29Nov22_test2$dominance = as.factor(Primary_analysis29Nov22_test2$dominance).

#Example of regression for age. Similar regressions were calculated to other variables.

m_outcome3 <—glmmTMB(outcome_1_exist ~ age_cat + gender_simple + experience + product_type + dominance + Pain_group + Mental_group + (1 | user), data = Primary_analysis29Nov22_test2, family = binomial).

summary(m_outcome3).

A3 <- confint(m_outcome3)

A3

exp(A3).

Anottry < -tidyr::tibble(exp(A3)).

write.csv(Anottry,file = "OR_all29Nov22.csv").

#Age:

p = ggplot(data = NewAge,

aes(x = Index,y = Estimate, ymin = LCI, ymax = UCI)) + geom_pointrange(aes(col = Index), show.legend = FALSE, fatten = 14) + xlab("Age") + ylab(("Odds ratios (95% Confidence Interval)")).

p + coord_flip() + 

scale_y_log10(breaks = c(0.25, 0.5,1,2,4),position = "left",limits = c(0.12,8.5)) + 

guides(col = guide_legend(reverse = TRUE)) + theme(plot.title = element_text(size = 12,face = "bold"),

axis.text.y = element_text(size = 10, face = "bold", vjust = 0.3),

axis.ticks.y = element_blank(),

axis.text.x = element_text(face = "bold"),

axis.title = element_text(size = 14,face = "bold"),

strip.text.y = element_text(hjust = 0,vjust = 1,angle = 180,face = "bold")) + geom_hline(yintercept = 1, linetype = "dashed",

color = "black", size = 0.5).

#Gender–- graph:

new_gender$Index <—factor(new_gender$Index, levels = c("males","females","other genders")).

p = ggplot(data = new_gender, aes(x = Index,y = Estimate, ymin = LCI, ymax = UCI)) + geom_pointrange(aes(col = Index), show.legend = FALSE, fatten = 14) + xlab("Gender") + ylab(("Odds ratios (95% Confidence Interval)")).

p + coord_flip() + 

scale_y_log10(breaks = c(0.33, 1,3),position = "left",limits = c(0.25,4)) + 

guides(col = guide_legend(reverse = TRUE)) + theme(plot.title = element_text(size = 12,face = "bold"),

axis.text.y = element_text(size = 10, face = "bold", vjust = 0.3),

axis.ticks.y = element_blank(),

axis.text.x = element_text(face = "bold"),

axis.title = element_text(size = 14,face = "bold"),

strip.text.y = element_text(hjust = 0,vjust = 1,angle = 180,face = "bold")) + geom_hline(yintercept = 1, linetype = "dashed",

color = "black", size = 0.5).

#Experience -graph:

New_experience$Index < -factor(New_experience$Index, levels = c("Experts","lot of experience","little experience","beginner")).

p = ggplot(data = New_experience,

aes(x = Index,y = Estimate, ymin = LCI, ymax = UCI)) + geom_pointrange(aes(col = Index), show.legend = FALSE, fatten = 14) + xlab("Experience in Cannabis use") + ylab(("Odds ratios (95% Confidence Interval)")).

p + coord_flip() + 

scale_y_log10(breaks = c(0.6, 0.8, 1.25, 1.8),position = "left",limits = c(0.4,2.5)) + 

guides(col = guide_legend(reverse = TRUE)) + theme(plot.title = element_text(size = 12,face = "bold"),

axis.text.y = element_text(size = 10, face = "bold", vjust = 0.3),

axis.ticks.y = element_blank(),

axis.text.x = element_text(face = "bold"),

axis.title = element_text(size = 14,face = "bold"),

strip.text.y = element_text(hjust = 0,vjust = 1,angle = 180,face = "bold")) + geom_hline(yintercept = 1, linetype = "dashed",

color = "black", size = 0.5).

#Reason provided–- graph.

New_indications$Index <—factor(New_indications$Index, levels = c("Other reasons","Pain","Mental")).

p = ggplot(data = New_indications,

aes(x = Index,y = Estimate, ymin = LCI, ymax = UCI)) + geom_pointrange(aes(col = Index), show.legend = FALSE, fatten = 14) + xlab("Reason provided") + ylab(("Odds ratios (95% Confidence Interval)")).

p + coord_flip() + 

scale_y_log10(breaks = c(0.8, 1.25),position = "left",limits = c(0.6,1.8)) + 

guides(col = guide_legend(reverse = TRUE)) + theme(plot.title = element_text(size = 12,face = "bold"),

axis.text.y = element_text(size = 10, face = "bold", vjust = 0.3),

axis.ticks.y = element_blank(),

axis.text.x = element_text(face = "bold"),

axis.title = element_text(size = 14,face = "bold"),

strip.text.y = element_text(hjust = 0,vjust = 1,angle = 180,face = "bold")) + geom_hline(yintercept = 1, linetype = "dashed",

color = "black", size = 0.5).

# composition–- graph:

p = ggplot(data = New_Composition,

aes(x = Index,y = Estimate, ymin = LCI, ymax = UCI)) + geom_pointrange(aes(col = Index), show.legend = FALSE, fatten = 14) + xlab("Product composition") + ylab(("Odds ratios (95% Confidence Interval)")).

p + coord_flip() + 

scale_y_log10(breaks = c(0.6, 0.8, 1.25, 1.8),position = "left",limits = c(0.4,2.5)) + 

guides(col = guide_legend(reverse = TRUE)) + theme(plot.title = element_text(size = 12,face = "bold"),

axis.text.y = element_text(size = 10, face = "bold", vjust = 0.3),

axis.ticks.y = element_blank(),

axis.text.x = element_text(face = "bold"),

axis.title = element_text(size = 14,face = "bold"),

strip.text.y = element_text(hjust = 0,vjust = 1,angle = 180,face = "bold")) + geom_hline(yintercept = 1, linetype = "dashed",

color = "black", size = 0.5).

# products.

new_products$Index <—factor(new_products$Index, levels = c("flowers","concentrates","tinctures","edible products","pills")).

p = ggplot(data = new_products,

aes(x = Index,y = Estimate, ymin = LCI, ymax = UCI)) + geom_pointrange(aes(col = Index), show.legend = FALSE, fatten = 14) + xlab("Type of product") + ylab(("Odds ratios (95% Confidence Interval)")).

p + coord_flip() + 

scale_y_log10(breaks = c(0.6, 0.8, 1.25, 1.8),position = "left",limits = c(0.4,2.5)) + 

guides(col = guide_legend(reverse = TRUE)) + theme(plot.title = element_text(size = 12,face = "bold"),

axis.text.y = element_text(size = 10, face = "bold", vjust = 0.3),

axis.ticks.y = element_blank(),

axis.text.x = element_text(face = "bold"),

axis.title = element_text(size = 14,face = "bold"),

strip.text.y = element_text(hjust = 0,vjust = 1,angle = 180,face = "bold")) + geom_hline(yintercept = 1, linetype = "dashed",

color = "black", size = 0.5).

p + coord_flip() + 

scale_y_log10(breaks = c(0.6, 0.8, 1.25, 1.8),position = "left",limits = c(0.5,2)) + 

guides(col = guide_legend(reverse = TRUE)) + theme(plot.title = element_text(size = 12,face = "bold"),

axis.text.y = element_text(size = 10, face = "bold", vjust = 0.3),

axis.ticks.y = element_blank(),

axis.text.x = element_text(face = "bold"),

axis.title = element_text(size = 14,face = "bold"),

strip.text.y = element_text(hjust = 0,vjust = 1,angle = 180,face = "bold")) + geom_hline(yintercept = 1, linetype = "dashed", color = "black", size = 0.5).

glm(symptom_difference ~  ~ age_cat + 

gender_simple + gender_simple_other + experience_little + 

experience_beginner + experience_lot + 

product_type_concentrate + product_type_edible + product_type_pill + 

product_type_tincture + dominance_T + dominance_C + Pain_group + 

Mental_group + (1 | user), data = all_groupsNov)).

##interaction age-gender – only Females vs males since not enough power for anayalsis with nonbinary.

maleFemale <—subset(Primary_analysis29Nov22_test2, gender_simple ! = 2).

m_outcome3MF <—glmmTMB(outcome_1_exist ~ age_cat + gender_simple + experience + product_type + dominance + Pain_group + Mental_group + (1 | user) + gender_simple*age_cat, data = maleFemale, family = binomial).

summary(m_outcome3MF).

## Appendix 2

List of cannabis-related events and reasons provided in the app for cannabis use.

The list of cannabis-related events includes all experiences and emotions listed in the app, and comprises of the case group and control group as follows: List of negative mental emotions included in the study (“case” group): anxious, confused, distracted, irritable, paranoid, scattered, restless.

List of emotions included in the study (“control” group): Active, Chill, Clear, Clumsy, Comfy, Couchlocked, Coughing, Creative, Dizzy, Dreamy, Dry Mouth, Energetic, Focused, Foggy, Forgetful, Frisky, Grateful, Great, Happy, Headache, High, Hungry, Light, Nausea, Optimistic, Peaceful, Productive, Rapid Pulse, Red Eyes, Reflective, Relaxed, Silly, Sleepy, Talkative, Thinky, Thirsty, Tingly, Tuned, Unmotivated, Visuals.

## Appendix 3

Directed acyclic graph (DAG) describing the relationship between studied variables and potential confounders in the study.

## Appendix 4

The list of the reasons provided in the app for cannabis use comprise of mental reasons, pain reasons and other reasons.

Mental reasons: Mental reasons provided for cannabis use include the following: Anxiety, Depression, Stress, Wellness,Fatigue,Agitation / Irritability, Insomnia, Hard to Focus Thoughts,Mood Swings, Restlessness, Hyperactivity, Aggression, Disassociation, Impulse,Oppositional Behaviors,Self Injurious Behaviors,Self Stimulatory Repetitive Behaviors,Language Difficulty.

Types of pain include in the reason provided for cannabis use include the following: Pain—Back, Pain—Headache, Pain—Joint, Pain—Muscle, Pain – Gastrointestinal, Pain – Nerve, Pain – Arm or Leg, Pain – Migraine, Pain – Abdominal, Pain – Cramping, Pain – Menstrual, Pain—Other.

Others: Constipation, Convulsions, Dizziness, Excessive Appetite, Eye Pressure, Hot Flashes, Inflammation, Joint Stiffness, Loss of Appetite, Low Libido, Muscle Spasms, Nausea, Numbness / Tingling, Other, Tinnitus, Tremors, Withdrawal—Alcohol, Withdrawal—Benzo, Withdrawal—Nicotine, Withdrawal—Opiate, Withdrawal—Other.

## Appendix 5

Explanation about the differences between the differences between the primary and secondary flow charts The secondary analysis required ranking of the symptom relief which was not available for all sessions as some participants did not report the severity before and after the use of cannabis. This led to a smaller initial pool of sessions: 75,557 sessions in the primary analysis compared to 65,553 sessions in the secondary analysis with full data (about 13% difference). However, the terms for duplicates exclusion were more restrictive in the secondary analysis as we also added the covariate of reported symptom releaf to the analysis, leading to the requirement for identical values in the following variables for exclusion: user’s ID, date of use, reason for use, and reported symptom relief. In the primary analysis, the requirement for identical values included only the first three abovementioned variables, i.e. user’s ID, date of use, and reason for use, leading to more duplicates exclusion.

## Appendix 6

No interactions were found between age and gender for reporting negative mental emotions.

**Table:**

Categories for interaction	p-value
Age 30–40 years*gender	0.75297
Age 40–50 years*gender	0.68572
Age 50–60 years*gender	0.59642
Age 60–70 years*gender	0.4345
Age 70–90 years*gender	0.87712
