# Supplementary material for: Characterization of Cannabis users and products and the experience of negative mental emotions following Cannabis use
Source: Eur Arch Psychiatry Clin Neurosci. 2024 Jun 11;275(2):407–20. doi: 10.1007/s00406-024-01812-0 (PMC11910409; doi:10.1007/s00406-024-01812-0)
Supplement: Supplementary file 1 — Supplementary file1 (DOCX 19 KB) [file 406_2024_1812_MOESM1_ESM.docx]

**Supplementary materials:**

Table S1: Demographic characterization of the users included population in the study

| Demographic characterization | | N | % out of (4,435) |
| --- | --- | --- | --- |
| Age group (Years) | 18-30 | 1,362 | 34.0 |
|  | 30-40 | 1,510 | 30.7 |
|  | 40-50 | 920 | 20.8 |
|  | 50-60 | 426 | 9.6 |
|  | 60-70 | 178 | 4.0 |
|  | 70-90 | 39 | 0.9 |
| Continent | North America (United states and Canada ) | 4,271 | 96.6 |
|  | Europe | 77 | 1.7 |
|  | Asia | 5 | 0.1 |
|  | America (American countries not including United States and Canada) | 59 | 1.3 |
|  | Africa | 11 | 0.2 |
|  | Australia | 12 | 0.3 |
| Gender | female | 2,583 | 58.2 |
|  | male | 1,697 | 38.3 |
|  | Other | 155 | 3.5 |
| Experience | beginner | 649 | 14.6 |
|  | little | 1,712 | 38.6 |
|  | lot | 1,549 | 34.9 |
|  | expert | 525 | 11.9 |

Table S2: Demographic characterization of cases (sessions with negative mental emotions)

| Characterization | | | N | % out of (10,664) |
| --- | --- | --- | --- | --- |
| Age group (Years) | | 18-30 | 2,893 | 27.1 |
|  |  | 30-40 | 3,553 | 33.3 |
|  |  | 40-50 | 2,333 | 21.9 |
|  |  | 50-60 | 1,303 | 12.2 |
|  |  | 60-70 | 488 | 4.6 |
|  |  | 70-90 | 94 | 0.9 |
| Continent | | North America (United states and Canada | 10,258 | 96.1 |
|  |  | Europe | 230 | 2.2 |
|  |  | Asia | 6 | 0.06 |
|  |  | America (American countries not including United States and Canada) | 75 | 0.7 |
|  |  | Africa | 39 | 0.4 |
|  |  | Australia | 56 | 0.5 |
| Gender | | Female | 6,274 | 58.8 |
|  |  | Male | 3,907 | 36.7 |
|  |  | Other | 483 | 4.5 |
| Experience | | Beginner | 1,590 | 14.9 |
|  |  | Little | 4,597 | 43.1 |
|  |  | A lot of experience | 3,329 | 31.2 |
|  |  | Expert | 1,148 | 10.8 |
| Product Type | | Concentrate | 3,813 | 35.8 |
|  |  | Edible | 846 | 7.9 |
|  |  | Flowers | 4,794 | 45.0 |
|  |  | Pills | 228 | 2.1 |
|  |  | Tincture | 983 | 9.2 |
| Dominance | | THC | 8,308 | 77.9 |
|  |  | Balanced | 1,037 | 9.7 |
|  |  | CBD | 1,319 | 12.4 |
| Reason provided | Pain | 2,372 | 22.2 |  |
|  | Mental reason | 5,202 | 48.9 |  |
|  | Other reason | 3,090 | 28.9 |  |
